# A Novel pH- and Salt-Responsive *N*-Succinyl-Chitosan Hydrogel via a One-Step Hydrothermal Process

**DOI:** 10.3390/molecules24234211

**Published:** 2019-11-20

**Authors:** Xingliang Li, Yihan Wang, Aoqi Li, Yingqing Ye, Shuhua Peng, Mingyu Deng, Bo Jiang

**Affiliations:** 1 College of Chemistry, Sichuan University; Chengdu 610064, China; 2 Jingkun Oilfield Chemistry Company; Kunshan, Jiangsu 215300, China

**Keywords:** *N*-succinyl-chitosan, glycidyloxypropyltrimethoxysilane, pH sensitivity, salt sensitivity, cross-linker

## Abstract

In this study, we synthesized a series of pH-sensitive and salt-sensitive *N*-succinyl-chitosan hydrogels with *N*-succinyl-chitosan (NSCS) and the crosslinker glycidoxypropyltrimethoxysilane (GPTMS) via a one-step hydrothermal process. The structure and morphology analysis of the NSCS and glycidoxypropyltrimethoxysilane-*N*-succinyl chitosan hydrogel (GNCH) revealed the close relation between the swelling behavior of hydrogels and the content of crosslinker GPTMS. The high GPTMS content could weaken the swelling capacity of hydrogels and improve their mechanical properties. The hydrogels show high pH sensitivity and reversibility in the range of pH 1.0 to 9.0, and exhibit on-off switching behavior between acidic and alkaline environments. In addition, the hydrogels perform smart swelling behaviors in NaCl, CaCl_2_, and FeCl_3_ solutions. These hydrogels may have great potential in medical applications.

## 1. Introduction

Hydrogels, as one of the most promising soft materials, have three-dimensional network structures composed of polymer and water [1,2,3,4]. Hydrogels with good environmental response have attracted more and more attention in pharmaceuticals, medicine, tissue engineering, materials science, food, and agriculture [5,6,7,8,9,10]. In particular, pH- and salt-responsive hydrogels are mostly studied because both parameters are important environmental factors in physiological and chemical systems [11,12]. Hydrogels made from natural polymers, including chitin [13], gelatin [14], cellulose [15], and sodium alginate [16], have many unique advantages, such as good biocompatibility, biodegradability, and these natural polymers have abundant resources. Natural polysaccharides, due to their unique advantages, can be used to make hydrogels for biomedical applications, such as stent coatings [17], especially in drug delivery [18].

Chitosan (CS), a biopolymer comprising glucosamine and *N*-acetylglucosamine, is an *N*-deacetylated product of chitin and the most abundant natural biomass material other than cellulose [19]. Chitosan has excellent biological properties such as biodegradability, biocompatibility, antibacterial, and wound healing [20,21,22]. However, the insolubility at neutral or high pH region has limited the application of chitosan. To improve the solubility of chitosan, a series of hydrophilic groups have been introduced into its skeleton, such as carboxymethyl chitosan [23,24], PEGylation [25], gallic acid grafting [26] etc. *N*-succinyl-chitosan (NSCS) is synthesized by attaching a succinyl group to the amine group of chitosan, which improves the solubility of chitosan in water. The pH-sensitive polymer made from NSCS is biocompatible and safe for human body [27].

The most common crosslinkers used to prepare chitosan-based hydrogels are dialdehydes such as glyoxal [28], and in particular glutaraldehyde [29,30]. However, they are mostly toxic [30,31]. The cytocompatible coupling agent glycidyloxypropyltrimethoxysilane (GPTMS) [32], has been conventionally applied in organic-inorganic hybrid materials via sol-gel reaction providing covalent linkage via the sol–gel reaction between organic and inorganic matrices. The representative sol–gel reaction is based on the silane functionality, silanol (Si-OH), ready for polycondensation to yield siloxane (Si-*O*-Si) bonds [33]. Therefore, GPTMS is an interesting alternative to prepare hydrogel.

Although a few studies have reported that chitosan and GPTMS are crosslinked to synthesize hydrogels [33,34], the cumbersome synthesis process and the harsh experimental conditions restrict the further application. In this work, *N*-succinyl-chitosan (NSCS) is synthesized from chitosan and succinic anhydride, and the glycidoxypropyltrimethoxysilane-*N*-succinyl chitosan hydrogel (GNCH) was prepared by one-step cross-linking reaction of NSCS with the crosslinker glycidoxypropyltrimethoxysilane (GPTMS). NSCS can completely dissolve in deionized water without further treatment and the synthesis process of hydrogel is mild and simple. GPTMS allows direct crosslinking reaction in aqueous media under mild conditions, and there is no addition of external molecules such as reducers which is of detrimental to biocompatibility. The synthesis and properties of the chitosan hydrogel are systematically studied and the results may provide a new approach for the preparation of smart-responsive hydrogels from natural biomass polymers. This kind of hydrogels may have great potential in the biomedical applications.

## 2. Results and Discussion

**Figure 1:**
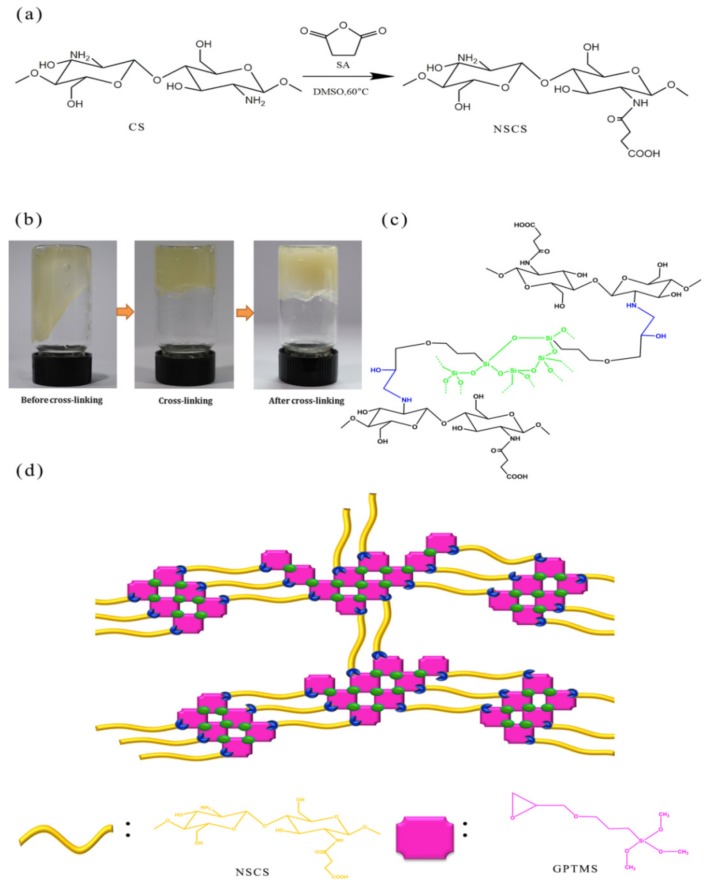
Synthesis scheme of the *N*-succinyl-chitosan hydrogel (GNCH). (**a**) Synthesis route of N-succinyl-chitosan (NSCS). (**b**) Gelation behavior of cross-linking hydrogel. (**c**) Scheme of cross-linking processes. (**d**) Schematic of the hydrogel structure.

The synthesis process of GNCH using NSCS is described in Figure 1. The formation mechanism of GNCH can be described as follows. The oxirane ring on the GPTMS reacted with the remaining amino group on the NSCS chain and hydration of the trimethoxy groups on the GPTMS formed silantriol pendent. Then the sol was heated at 80 °C to form inter-chain linkages between NSCS chains via the dehydration reaction among the silantriol groups. The reaction units are marked with green and blue, respectively.

### 2.1. Structural Characterization

**Figure 2:**
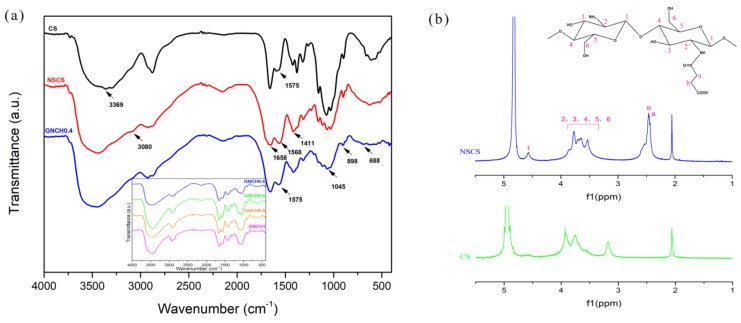
(**a**) FTIR spectra of chitosan (CS), *N*-succinyl-chitosan (NSCS), GNCH. Inset: FTIR spectra of GNCH (0.4–1). (**b**) Structural formula and ^1^H NMR spectra of CS and NSCS.

Figure 2a shows the FTIR spectra of CS, NSCS and GNCH. For the CS, the absorption peak located at 1575 cm^−1^ is attributed to the -NH_2_ bending vibration. The absorption peak located at 3369 cm^−1^ is assigned to the -OH stretching vibration, and the absorption peaks located at 3030–3330 cm^−1^ are ascribed to the –NH_2_ stretching vibration. No absorption peaks at 3080 cm^−1^ is observed in the infrared spectrum of CS due to the intramolecular and intermolecular hydrogen bonds. For the NSCS, two new characteristic absorption peaks appear at 1658 cm^−1^ and 1411 cm^−1^ correspond to the formation of -CO-NH- [35], and the obvious absorption peaks at 3080 cm^−1^ indicate the -NH_2_ of CS has been partially substituted by succinyl groups (-NH(CO)-CH_2_-CH_2_-COOH), converting the primary amines (-NH_2_) into secondary amides [36].

In the spectrum of the NSCS, the absorption peaks at 1568 cm^−1^ is attributed to the N–H absorption [37].40 In GNCH, the intensities of the peak at 1575 cm^−1^ decreased are assigned to the N–H formed after cross-linking. The peak of 1045 cm^−1^ and 688 cm^−1^ are attributed to the Si-*O*-Si symmetrical stretching vibration and the asymmetric stretching vibration peak of Si-*O*-Si, respectively. The peak of 898 cm^−1^ corresponds to the Si-OH bond [38]. The FT-IR results confirm that GPTMS has successfully cross-linked with NSCS.

Chemical structure and ^1^H NMR spectra of *N*-succinyl-chitosan are shown in Figure 2b. The peak at 4.57 ppm is ascribed to H-1 of glucosamine (GlcN), and the peak at 3.54–3.86 ppm is ascribed to H-2, H-3, H-4, H-5, H-6 of GlcN and H-2′ of *N*-acylated GlcN. Furthermore, the peak at 2.45 ppm (H-a) and 2.46 ppm (H-b) correspond to -NH(CO)-CH_2_- and -CH_2_-COOH of the substituted succinyl group (-NH(CO)-CH_2_-CH_2_-COOH), respectively [35]. The degree of substitution (DS) is calculated using Equation (1):

(1) $$\mathrm{DS}\;=\;\frac{A^{\prime }\times \frac{1}{4}}{A^{\prime \prime }\times \frac{1}{6}}\;\times \;100\%$$

where the A′ represents the integral value of protons corresponding to -CH_2_-CH_2_- (H-a and H-b) of the substituted succinyl group (-NH(CO)-CH_2_-CH_2_-COOH), and the A″ represents the integral value of protons corresponding to H-2, H-2′, H-3, H-4, H-5 and H-6 [39]. The calculated value of DS is 71%. Compared with literature [40], the degree of substitution of NSCS was further improved, which was conducive to the complete dissolution of NSCS in distilled water.

Therefore, the FTIR spectra together with ^1^H NMR spectra indicate that the successful preparation of NSCS and the FTIR spectra indicate the successful preparation of GNCH.

### 2.2. SEM Analysis

**Figure 3:**
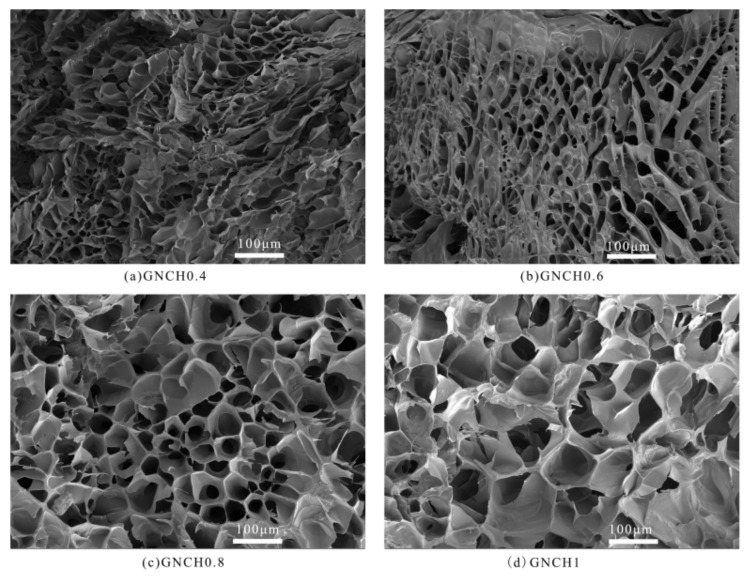
SEM images of hydrogels (**a**) GNCH0.4, (**b**) GNCH0.6, (**c**) GNCH0.8, (**d**) GNCH1.

Figure 3 shows the interior morphological structures of freeze-dried GNCH with different GPTMS contents. All the hydrogels display a continuous and porous three-dimensional structure, which is caused by phase separation and sublimation of removing water during the freeze-drying process [41]. In addition, the pore size of hydrogel became larger as GPTMS contents increased. The reason is that the increased cross-link density could cause faster phase separation during freezing, resulting in a large pore size phase structure [42].

### 2.3. Swelling Properties

**Figure 4:**
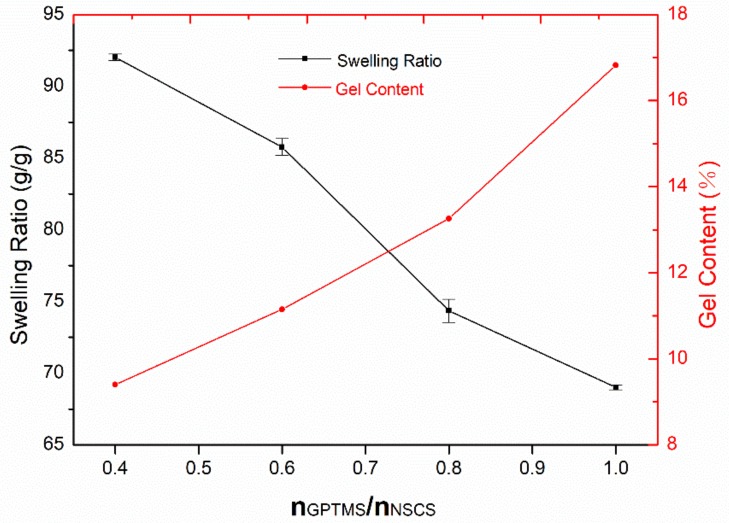
Swelling ratio (g/g) and gel content (G%) of GNCH.

As reported, the swelling capacity of the hydrogel decrease with the increase of crosslinker concentration. It can be seen from Figure 4, as the molar ratio of GPTMS to NSCS increased from 0.4 to 1, the swelling ratio of hydrogel decreased from 92 to 69 g/g, which makes it have good application in biomedicine. Meanwhile, the gel content increased with the increase of GPTMS content. So we can presume that the decrease in swelling ratio is associated with the increase in cross-link density of the gel.

**Figure 5:**
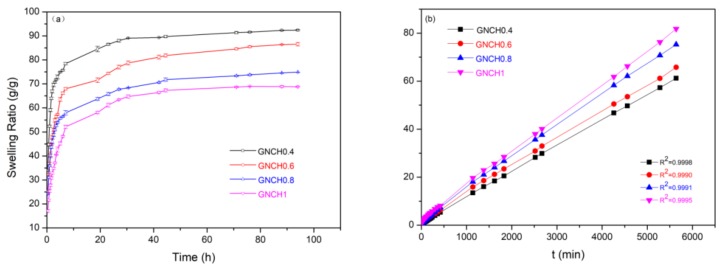
(**a**) Swelling rate and (**b**) pseudo-second-order kinetics of hydrogels in deionized water. All the values of correlation coefficient (R^2^) ≥ 0.999.

Figure 5 shows the time function of hydrogel swelling ratio. The swelling behavior of GNCH in distilled water is related to the content of crosslinker. The amount of absorbed water increased rapidly during the initial swelling for each hydrogel and then slowed down until reaching equilibrium at about 70 h. This behavior is analyzed using a second-order swelling kinetics model (Equation (2)) [43].

(2) $$\frac{t}{{SR}_{t}}=\frac{1}{{K\cdot \mathrm{SR}}_{eq}^{2}}+\frac{t}{{\mathrm{SR}}_{\mathrm{eq}}}$$

where *SR_t_* is the swelling ratio at given swelling time *t* (s); K is the swelling rate constant; SR_eq_ is the swelling ratio at equilibrium time [41]. The *t*/*SR_t_* is linear with t and its correlation coefficient is greater than 0.999 (Figure 5b), which accords with the second-order swelling kinetics model [44].

According to Figure 5b, the swelling rate constant (K) and the experimental values of swelling ratio (SR_eq_) were obtained from the experiment data, listed in Table 1. The minimum swelling ratio of the hydrogels and the lowest swelling rate constant (K) were obtained at the most cross-linked GNCH1, while the maximum swelling ratio of the hydrogels was obtained at the least cross-linked GNCH0.4. A similar phenomenon was also previously noted in the study of another hydrogel material [41]. This result was likely due to an increase in crosslinking density as the amount of the crosslinker increases, resulting in a decrease in the swelling ratio of the hydrogel. These results indicate that the increase of GPTMS content will increase the crosslinking density of GNCH, the swelling ratio of the hydrogel is inversely proportional to the amount of crosslinker GPTMS [44].

### 2.4. pH-Sensitive Behavior

**Figure 6:**
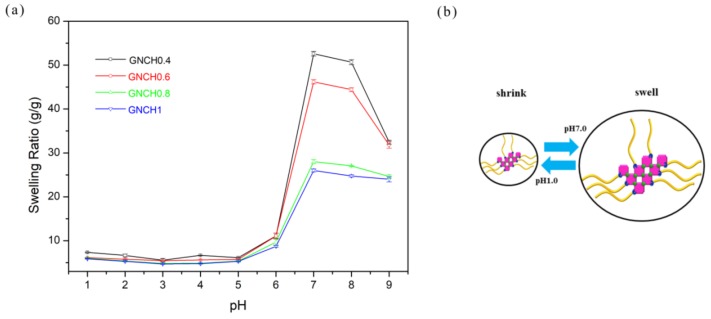
(**a**) Variation of swelling capacity for GNCH at the buffer solution with various pH values. (**b**) An illustration of the size comparison of GNCH at different pH values.

The pH-responsive behaviors of hydrogels from pH = 1.0 to pH = 9.0 are presented in Figure 6. The ionic strength of various pH solutions was controlled at 0.4 M by adjusting NaCl content. All hydrogels exhibited lower swelling ratio in 0.4 M ionic strength buffers compared with that in distilled water. Four samples of the GNCH exhibited clearly pH-sensitive behavior in buffers, which obtained the maximum swelling ratio at pH = 7.0. The volume of GNCH changed in a wide range of pH value due to acidic groups. The different pH-depended interacting species in swelling medium lead to the change of equilibrium swelling ratio (SR). Therefore, based upon pK_a_ of SA (4.19) and pK_b_ of CS (6.5), the involving species are mainly −COOH at pH 1.0–6.0, and −COO− at pH 7.0–9.0. At low pH (<7.0), because of the strong acidic condition, the dominant charges in the gels are acid form (−COOH); and at high pH (7.0–9.0), the dominant charges are the ionised carboxyl groups (−COO−).

When at pH 1.0–6.0, the acid form (−COOH) could form intermolecular hydrogen bonds, which resulted in the unfavorable swelling behavior and lower swelling ratio for hydrogels. At pH = 7.0, the carboxyl groups gradually transformed into the ionized carbonate form (−COO−), leading to stronger hydrophilicity and higher electrostatic repulsion of the network, and hence enhance the water absorption capacity [45]. However, the repulsion of the negative −COO− groups would be shielded by more Na^+^ in the basic condition (pH > 7.0) for screening effect, causing the shrinking of hydrogels, thus their swelling ratio decreased subsequently. The ionic groups play the main role in swelling variations of the GNCH. These results suggest that the swelling behavior of GNCH can be controlled by varying pH of the solution [46].

### 2.5. pH Reversible Behavior

**Figure 7:**
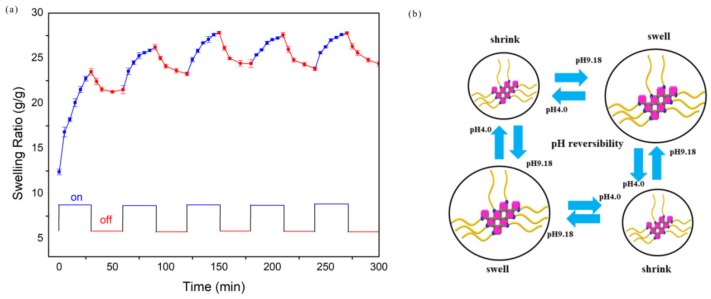
(**a**) On-off switching behavior as reversible pulsatile swelling (pH 9.18) and deswelling (pH 4.0) of GNCH. The time interval between pH changes is 30 min; (**b**) Mechanism of pH reversible process.

The pH-responsive behavior of GNCH was demonstrated to be reversible. Figure 7a shows a stepwise reproducible swelling change of the hydrogels with alternating pH between 4.0 and 9.18, demonstrating a reversible pH-responsive behavior of GNCH. The mechanism of the pH reversible effect is explained as showed in Figure 7b. The hydrogels reach higher swelling ratio at pH 9.18, but the swollen gel rapidly shrink due to the protonation of –COO− groups and exhibit intriguing on–off switching behavior [44], while at pH 4.0, the hydrogels shrink within a few minutes due to protonation of carboxylate groups [47].

After five cycles, the hydrogels exhibit well swelling-deswelling performance, which makes them suitable candidates for controlled drug delivery systems [48]. The evident change of water absorption with altering the pH of external buffer solution confirms the excellent pH-sensitive characteristic of GNCH.

### 2.6. Salt Sensitivity Behavior

**Figure 8:**
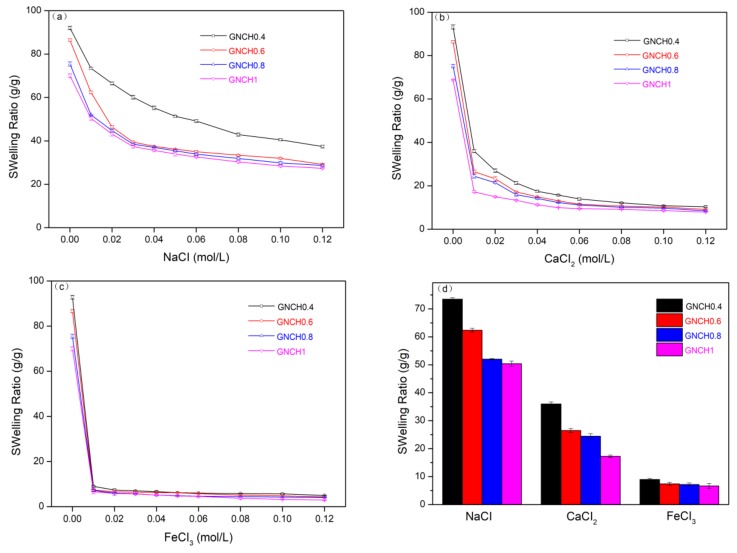
Swelling variation of GNCH in (**a**) NaCl solution, (**b**) CaCl_2_ solution, (**c**) FeCl_3_ solution, (**d**) Swelling ratio of hydrogels in different salt solutions (0.01 M): NaCl, CaCl_2_, and FeCl_3_.

The swelling behavior of the GNCH in various salt solutions is shown in Figure 8. In general, the salt-sensitive hydrogel consists of three phases, namely the three-dimensional polymeric network matrix, the interstitial fluid, and the ionic species [49]. In NaCl, CaCl_2_, and FeCl_3_ solutions, a marked volume decrease was observed in hydrogels with the increasing of salt concentration, the swelling ratio of gels in saline solutions was appreciably reduced comparing to the values measured in deionized water. The swelling and shrinking behaviors of hydrogels in salt solution were determined by the ionic interactions between mobile ions and the fixed charges which make tremendous contributions to the osmotic pressure between the interior hydrogel and external solution. Because of the Donnan osmotic pressure, the gels began to shrink in higher salt concentrations [50].

The swelling ratio of GNCH exhibited sharp decrease with an increase of salt concentration in CaCl_2_ and FeCl_3_ solution, as shown in Figure 8b, c. The higher cation charges lead to higher degree of crosslinking and the smaller swelling value. Because of the swelling ratio of hydrogels in salt solution depended not only on the salt concentration but also on the ionic charge. Figure 8d shows the swelling ratio of the hydrogels with different proportion of crosslinkers in various salt solutions (0.01 M). Under the presence of excess salt, the counterion contribution to the osmotic pressure increased with the increasing of ionic charge. The higher cation charges lead to form internal or intermolecular complexes of −COO− groups inside the gel, and a multivalent ion can neutralize several charges within the gel. Consequently, the crosslinking density of the network increases, while the water absorption capacity decreases. Therefore, the swelling ratio of the hydrogel in the studied salt solutions is in the order of monovalent > divalent > trivalent cations [47].

### 2.7. Rheological Properties

**Figure 9:**
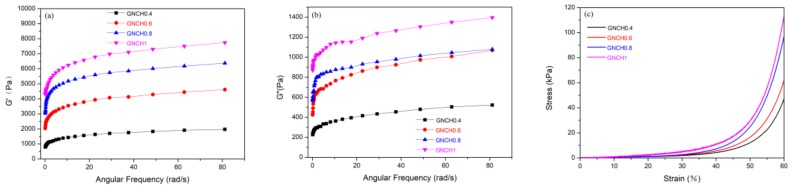
(**a**) Storage modulus (G’, 1% strain) and (**b**) loss modulus (G”, 1% strain) as a function of frequency (Hz) at 25°C. (**c**) Compressive stress-strain curves at 60% strain for GNCH with various cross-linker contents.

Figure 9 shows the rheological properties of GNCH with different proportions of GPTMS at 25 °C. The gels exhibited typical viscoelastic behavior, as both the storage modulus (G’; Figure 9a) and loss modulus (G”; Figure 9b) increased with oscillating frequency. G’ was larger than G” over the whole range of frequency, suggesting a general dominance of the elastic response of the gels to deformation over a broad time scale. G’ of all GNCH was higher than G” over the whole selected angular frequency range [51]. Besides, both G’ and G” showed a monotonous increase with GPTMS content in the gels, which was probably due to the improvement in the network structure of these samples and increased cross-link density [42,52]. Moreover, the consequence of higher cross-link density of the gel lead to more heat dissipation for chain segment movement [41]. The positive effect of GPTMS content on the mechanical properties of GNCH could also be observed in their compressive stress−strain curves (Figure 9c), where GNCH1 presented much higher stress values than the other hydrogels over the entire examined strain range. The storage modulus (G’) and loss modulus (G”) together with the compressive stress−strain curves show that the mechanical properties of GNCH can be significantly improved by increasing the content of GPTMS. Typically, the mechanical properties and rheological properties of chitosan hydrogels in recent related studies are listed in Table 2. Apparently, the mechanical property and preparation method of GNCH in our work are good and simple, which can be useful in design of new chitosan hydrogel.

## 3. Materials and Methods

### 3.1. Materials

Chitosan (CS, MW ≥ 3$\;{\times \;10}^{6}$, deacetylation degree of 84%) was purchased from Sigma-Aldrich (Missouri, USA). Succinic anhydride (SA, purity 99%), was purchased from Beijing InnoChem Science&Technology Co.Ltd, Beijing, China. Glycidoxypropyltrimethoxysilane (GPTMS, purity 98%) was obtained from Chengdu Kelong Chemical Co. Ltd., Chengdu, China. All the reagents were used as received without any further purification. Deionized water was used throughout.

### 3.2. Synthesis of N-succinyl-chitosan (NSCS)

Chitosan (5 g) was dissolved in 100 mL DMSO, then succinic anhydride (2.29 g) was added under stirring at 500 rpm for 4 h at 60 °C. The pH of the mixture after reaction was adjusted to 7 with 5% (*w*/*v*) NaOH (3 mL). After filtration, the precipitate was dissolved in 400 mL distilled water to prepare a solution of pH = 11 with 5% (*w*/*v*) NaOH (47 mL). This solution was recrystallized from acetone to form the pale yellow solid, and then washed with 400 mL of 75% acetone, 400 mL of 70% ethanol, and 400 mL of acetone, sequentially. The final product was dried under vacuum at 60 °C for 48h to obtain *N*-succinyl-chitosan (NSCS) particles [35]. The calculated yield of NSCS is 90.81%.

### 3.3. Synthesis of Glycidyloxypropyltrimethoxysilane-N-Succinyl-chitosan Hydrogels (GNCH)

GNCH were prepared by one-step hydrothermal process. A 8% (*w*/*v*) solution of NSCS in distilled water was prepared, and then mixed with a given amount of GPTMS for stirring at 100 rpm with 10 min to obtain a homogeneous solution. The reaction was let to proceed at 80 °C for 48 h. The five samples were labeled as GNCH0.4, GNCH0.6, GNCH0.8, GNCH1 by changing the molar ratio of GPTMS to NSCS to 0.4, 0.6, 0.8, 1. The hydrogel was extracted, cut into pieces and immersed in distilled water to remove the residual reactants and obtain pure samples. The washed hydrogel was dried for 48 h in a freeze dryer and used in the experiment. Figure 1 shows the hydrogel formation mechanism.

### 3.4. Fourier Transform Infrared Spectroscopy (FTIR)

FTIR spectroscopy of dry gel samples were conducted on a Bruker Tensor 27 FT-IR spectrometer (Karlsruhe, Germany) using KBr pellets and collected ranging from 4000 to 400cm^−1^.

### 3.5. Nuclear Magnetic Resonance (NMR)

^1^H-NMR spectrum of CS and NSCS samples were obtained in D_2_O at 25 °C with Bruker AV II-600 MHz (Bruker, Zurich, Switzerland).

### 3.6. Scanning Electron Microscope (SEM)

The swollen hydrogels with different proportions of crosslinker (0.4–1) were freeze-formed under liquid nitrogen and then freeze-dried. The freeze-dried hydrogel was examined by surface-coated with Au. The cross-sections of the lyophilized samples were visualized using a scanning electron microscopy (SEM, Hitachi S-4800, Tokyo, Japan).

### 3.7. Gel Content

The gel content (G%) [41] is calculated according to Equation (3):

(3) $$G\%\;=\;\frac{W_{a}}{W_{b}}\;\times \;100$$

where the *W*_a_ represents the weight of the dried hydrogel (washed), and *W*_b_ represents the weight of unwashed hydrogel.

### 3.8. Swelling Behaviors of Hydrogel

The swelling studies of the GNCH were carried out by the following method. All hydrogels were cut into 10 mm × 15 mm length (5 mm in thickness). The swelling ratio of hydrogels were studied by gravimetric method. The hydrogels were immersed in the distilled water, different pH solutions, and salt solutions at 25 °C for 4 days to reach equilibrium. Adjusting the pH value from 1 to 9 with Na_2_HPO_4_•12H_2_O, NaH_2_PO_4_•2H_2_O, C_6_H_8_O_7_, KCl, HCl, Na_2_CO_3_, and NaHCO_3_. The ionic strength of the pH solutions was 0.4 M, which was obtained by adding an appropriate amount of NaCl. The equilibrium swelling ratio (SR) of the hydrogel is calculated using Equation (4):

(4) $$\mathrm{SR}\;\left(g/g\right)\;=\;\frac{W_{s}\;-\;W_{d}}{W_{d}}$$

where the *W*_s_ and *W*_d_ represents the weight of swollen gel and dry gel, respectively. Three replicates were conducted to determine the average SR value of each sample.

### 3.9. Rheological Measurement Test

The sample was subjected to a rheological test using HAAKE Rheowin MARS III (HAAKE, Karlsruhe, Germany). The hydrogel sample was first cut into a cylinder with height of 1 mm and diameter of 25 mm, and then placed in a 25 mm flat geometry. The storage modulus (G’) and the loss modulus (G’’) were measured from 0 to 80 rad/s at 25 °C, 1% strain. The samples used in the compression test were cylindrical with a diameter of 14 mm and a height of 16 mm, and the compression rate was kept at 2 mm/min.

## 4. Conclusions

In summary, pH-sensitive and salt-sensitive *N*-succinyl-chitosan hydrogel (GNCH) can be prepared with NSCS and the crosslinker GPTMS. GNCH exhibit excellent pH-sensitive and pH reversibility due to the carboxyl from chitosan moieties. Study of swelling kinetics reveals that the pseudo-second-order model is suitable for illustrating the water absorption behavior of GNCH. Furthermore, hydrogels perform smart swelling behaviors in NaCl, CaCl_2_, and FeCl_3_ aqueous solutions, and their swelling ratio decrease with an increase of the salt concentration. Rheological properties of GNCH increase with GPTMS contents in the polymeric network. This work offers an efficient and practical way to prepare smart-responsive hydrogels from chitosan. These smart hydrogels can have wide applications in the fields of agriculture, foods, and tissue engineering.

## Figures and Tables

**Table 1:** Parameters obtained from swelling kinetics.

Samples	K [g/(g•min)]	SR_eq_ (g/g)
GNCH0.4	38 × 10^−5^	92.02
GNCH0.6	16 × 10^−5^	85.76
GNCH0.8	12 × 10^−5^	74.35
GNCH1	7.0 × 10^−5^	69.02

**Table 2:** The storage modulus of CNS5 [53], the storage modulus of CSMA3/SC3 [54], the storage modulus of Ch-3% NP [55], the Compressive strength and storage modulus of TNC200 (25 °C) [56], the Compressive strength and storage modulus of SHC0.075BGP0.1 [57], the Compressive strength and storage modulus of GNCH1.

Materials	Method	Compressive Strength (kPa)	Storage Modulus (Pa)	Year (ref.)
citroaromatic compounds/chitosan (CNS5)	heterogeneous reaction method/ nitrogen atmosphere	/	≈850	2019 (ref53)
NSCS/chondroitin sulfate multiple aldehyde (CSMA3/SC3)	Schiff base reaction	/	≈7000	2015 (ref54)
chitosan/poly(lactic-*co*-glycolic acid) (Ch-3% NP)	sol-gel	/	≈1000	2018 (ref55)
Chitosan/Poly (*N*-isopropylacrylamide) (TNC200)	free radical grafting polymerization/initiator and catalyst	≈15	≈5000	2018 (ref56)
chitosan (SHC0.075BGP0.1)	Injectable chitosan hydrogel	≈70	≈5000	2019 (ref57)
NSCS/GPTMS (GNCH1)	one-step hydrothermal process	≈110	≈7700	This work
